# Assessment of the impact of CT scan protocols on carbon ion radiotherapy treatment planning

**DOI:** 10.1002/acm2.70203

**Published:** 2025-08-21

**Authors:** Yuya Miyasaka, Hikaru Souda, Yasuhito Hagiwara, Hiroko Akamatsu, Mayumi Harada, Takashi Kaneko, Hongbo Chai, Miyu Ishizawa, Hiraku Sato, Masashi Koto, Takeo Iwai

**Affiliations:** ^1^ Department of Heavy Particle Medical Science Yamagata University Graduate School of Medical Science Yamagata Japan; ^2^ Department of Radiation Oncology Yamagata University Faculty of Medicine Yamagata Japan; ^3^ Department of Radiology Nihon‐kai General Hospital Yamagata Japan

**Keywords:** carbon‐ion therapy, CT scan protocol, CT‐SPR conversion table, dose evaluation, treatment planning

## Abstract

**Purpose:**

The purpose of this study was to quantify the impact of computed tomography (CT) scan protocols and CT scanner on carbon ion radiotherapy treatment planning

**Material and Methods:**

A table was created for each of the five parameters to relate CT values to stopping power (SPR) (CT‐SPR conversion table). The parameters changed in the four protocols, with the exception of the clinical protocol, are as follows: tube voltage, convolution kernel, CT scanner, and CT model. Treatment plans were calculated with each of the five CT‐SPR conversion tables applied, and differences from the doses in the clinical protocol were evaluated by comparison of DVH parameters and gamma analysis.

**Results:**

The largest difference in CT values in the protocols with changing tube voltage compared to the clinical protocol was more than 200 HU for the high CT value material. The change in CT values due to the change in the reconstructed kernel was slight, and the difference in CT values for the CT scanner change or model change protocol was similar. Mean difference <1% for pelvic region plans for changes in DVH parameters. The difference in DVH parameters between CT scan protocols for treatment planning in the head and neck region compared to the pelvic region was large, with a maximum difference of 6.17%. Gamma analysis showed that a 3%/3mm tolerance resulted in a near 100% pass rate for both CT scan protocols, while 2%/2mm and 1%/1mm resulted in a 5%–10% or greater pass rate reduction.

**Conclusion:**

The effect of changes in CT‐SPR conversion table due to changes in CT scan protocols on carbon ion radiotherapy treatment planning was quantitatively evaluated, and the importance of calibration for each CT scan protocol in carbon ion radiotherapy was clarified.

## 1 INTRODUCTION

Recently, favorable outcomes have been reported for carbon ion radiotherapy (CIRT), which plays an important role in cancer treatment.^1^, ^2^, ^3^, ^4^, ^5^, ^6^, ^7^ Accurate estimation of the irradiated dose in the body through treatment planning is important for CIRT as it is for X‐ray and proton therapy. Information on the stopping power ratio (SPR) of tissue materials is necessary to estimate not only the particle range in the body but also the absorbed dose in the media itself.^8^, ^9^, ^10^ In the current treatment planning protocols for CIRT, obtaining the SPR of each tissue from computed tomography (CT) images is common practice. The relationship between SPR and Hounsfield Unit (HU) is registered in the treatment planning system as a CT‐SPR conversion table, which is used for particle range and dose calculations. Schneider et al. proposed a method for estimating the SPR from HUs.^11^ They noted difficulties in applying the commercial phantom material to particle therapy because it is not tissue equivalent. In contrast, Kanematsu et al. proposed a method based on a polybinary tissue model using tissue substitute materials and a simple method for estimating the SPR.^12^, ^13^ Their proposed method has been adopted and used in clinical treatment planning at various facilities, including those in Japan.^10^ The premise of these methods is to calibrate for differences in CT scan protocols and CT scanner. Differences in CT‐SPR conversion tables may occur when scan protocols or CT scanners are changed, which may result in differences in treatment planning. Taasti et al. investigated variations in CT‐SPR conversion tables according to the particle therapy facility.^9^ They noted differences not only in the CT scanner used at each facility but also in the tube voltage and convolution kernels. In X‐ray therapy, the influence of differences in HUs in treatment planning due to differences in CT scan protocols was reported. For example, Cozzi et al. reported that the effects of changes in HUs were <2% even up to 300 HU,^14^ and Thomas et al. reported that the maximum dose error was <1% within the range of possible errors.^15^ Regarding the direct density method for relating CT‐SPR conversion in proton therapy, Yasui et al. reported on the effects of tube voltage and convolution kernel.^16^ In contrast, the effects of differences in CT scan protocols on treatment planning have not been quantitatively evaluated for CIRT. The dose distribution of CIRT is steeper than that of X‐rays or protons.^17^, ^18^ This may result in large unacceptable dose differences in CIRT, even small differences that are tolerated in x‐ray or proton therapy. For this reason, CIRT needs to independently evaluate the impact of the CT‐SPR conversion table. In this study we aim to evaluate the robustness with respect to CT‐SPR conversion tables. Although CT imaging protocol should be consistent, confirming the robustness of the CT‐SPR conversion table to changes in CT scanner output, CT scanner replacement, and even the use of multiple CT scanner at a facility can provide significant insight into the treatment stability of CIRT. Therefore, this study evaluated the effects of different scanning protocols and CT scanners on the CT images of the CIRT treatment plan.

## 2 MATERIAL AND METHODS

### 2.1 Creation of CT‐SPR conversion tables for each CT scan protocol

In this section, how to create a CT‐SPR conversion table is described. The creation of a CT‐SPR conversion table was based on the method reported by Kanematsu et al.^12^, ^13^ This method obtains the HUs of four materials (i.e., air, 100% ethanol, water, and 40% K_2_HPO_4_ solution) that are substitute materials for human body tissue, from which the CT values of the 11 tissues of the human body were estimated. H, the HU of the 11 tissues, was calculated by the following Equation (1) according to Kanematsu et al.^13^

(1) $$H=1001\left(\frac{Z_{ph}^{3.62}k_{ph}+Z_{coh}^{1.86}k_{coh}+1}{1487.1k_{ph}+38.463k_{coh}+1}n_{e}^{\prime }-1\right)$$

The parameters $k_{ph}$ and $k_{coh}$ in the x‐ray model in Equation (1) were calculated by the following Equations (2) and (3).

(2) $$k_{ph}=\frac{1}{1487.1}\;\frac{0.20484\mu_{K}^{\prime }+1.3514\mu_{E}^{\prime }-1.3578}{0.10139\mu_{K}^{\prime }+5.2635\mu_{E}^{\prime }-3.3392}$$

(3) $$k_{coh}=\frac{1}{38.463}\;\frac{5.7101-0.30623\mu_{K}^{\prime }-6.6149\mu_{E}^{\prime }}{0.10139\mu_{K}^{\prime }+5.2635\mu_{E}^{\prime }-3.3392}$$

The $\mu_{K}^{\prime }$ and $\mu_{E}^{\prime }$ in Equations (2) and (3) were calculated by Equation (4) using the HUs of $H_{w}$, $H_{a}$, $H_{E}$, and $H_{K}$ for water, air, 100% ethanol, and 40% K_2_HPO_4_ solution, respectively.

(4) $$\mu_{E,K}^{\prime }=\frac{H_{E,K}-H_{a}}{H_{w}-H_{a}}$$

The $Z_{ph}$ and $Z_{coh}$ in Equation (1) is the value shown in the report by Kanematsu et al.^13^ A CT‐SPR calibration phantom of the same type was used as a phantom, as reported by Kusano et al.^19^ To compensate for differences in subject size, a large phantom and a small phantom were used for data acquisition of the body and head and neck region images, respectively. Each phantom was equipped with two syringes, each containing four materials. CT images of the phantom were acquired using the five CT protocols and CT models shown in Table 1. First, the protocols were identical to those of clinical use (Body CL and Head CL). These protocols were used in clinical treatment planning as validated by Miyasaka et al.^10^ The second and third protocols were changed from Body CL and Head CL, respectively, to tube voltage (Body MV and Head MV) and convolution kernel from FC13 to FC09 (large phantom) and FC64 (small phantom) (Body CK and Head CK). FC64 and FC09 used a kernel for beam hardening correction, compared with FC13.^20^, ^21^ The fourth protocol was to change the CT scanner from Aquilion One (Canon Medical systems, Otawara, Japan) to Aquilion LB (Canon Medical systems, Otawara, Japan) under clinical scan protocols (Body LB and Head LB). Aquilion One had 320‐row detectors (Canon Medical systems, Otawara, Japan),^22^ whereas Aquilion LB had 16‐row detectors (Canon Medical systems, Otawara, Japan). In the fifth protocol, data were acquired on different models of the same CT scanner (Body MD and Head MD). Different models mean devices with the same name, Aquilion One, but different generations of the model and minor changes in the internal structure. Specifically, TSX‐305A was used for the body CL and head CL, and TSX‐301A for the body MD and head MD. The CT value of each material in each syringe of the CT image acquired under each protocol was measured. The diameter of the syringe in the phantom was 2 cm, and the region of interest (ROI) for obtaining HUs was a 1.3 cm diameter circle. An ROI was set at the center of the syringe at the CT origin, and the average HU within that ROI was estimated.

**TABLE 1:** Contents of the CT scan protocol.

Protocol name	Tube voltage (kV)	Convolution kernel	CT Scanner Name	CT Model Name
Body CL	120	FC13	Aquilion One	TSX‐305A
Body MV	80	FC13	Aquilion One	TSX‐305A
Body CK	120	FC64	Aquilion One	TSX‐305A
Body LB	120	FC13	Aquilion LB	TSX‐201A
Body MD	120	FC13	Aquilion One	TSX‐301A

Protocol Name	Tube voltage (kV)	Reconstraction kernel	CT Scanner Name	CT Model Name
Head CL	120	FC13	Aquilion One	TSX‐305A
Head MV	80	FC13	Aquilion One	TSX‐305A
Head CK	120	FC09	Aquilion One	TSX‐305A
Head LB	120	FC13	Aquilion LB	TSX‐201A
Head MD	120	FC13	Aquilion One	TSX‐301A

**TABLE 2:** Hounsfield Units for the four materials in the CT‐SPR calibration phantom for each CT scan protocol.

	Protocol				
Material	Body CL (HU)	Body MV (HU)	Body CK (HU)	Body LB (HU)	Body MD (HU)
Air	−960.1 ± 2.3	−954.8 ± 2.7	−950.1 ± 0.4	−1001.2 ± 0.1	−976.0 ± 1.3
Ethanol	−197.9 ± 0.2	−208.4 ± 2.5	−197.0 ± 0.7	−224.1 ± 1.4	−206.4 ± 0.6
Water	3.6 ± 0.9	4.5 ± 2.6	2.7 ± 1.3	−15.5 ± 0.2	−4.3 ± 2.8
K_2_HPO_4_ 40%	617.3 ±0.2	844.7 ± 0.3	613.8 ± 4.3	662.4 ± 2.6	649.7 ± 0.4

Material	Head CL (HU)	Head MV (HU)	Head CK (HU)	Head LB (HU)	Head MD (HU)
Air	−980.8 ± 2.6	−979.7 ± 4.7	−970.9 ± 13.3	−1030.0 ± 3.9	−989.8 ± 3.0
Ethanol	−205.4 ± 1.3	−217.2 ± 0.9	−203.8 ± 0.8	−235.2 ± 0.5	−211.7 ± 1.1
Water	5.6 ±1.4	8.1 ± 1.3	7.5 ± 1.1	−13.0 ± 2.1	−0.2 ± 0.4
K_2_HPO_4_ 40%	666.9 ± 4.1	897.2 ± 3.8	671.4 ± 8.9	740.5 ± 7.4	720.6 ± 0.3

Abbreviations: CT, computed tomography; HU, Hounsfield unit.

### 2.2 Adaptation and evaluation of the CT‐SPR conversion table

The impact of CT scan protocols and CT scanners on treatment planning was evaluated by comparing the calculated doses on CT images with each protocol CT‐SPR conversion table. First, the Body CL and Head CL CT‐SPR conversion table was applied to the treatment planning CT images, and the dose was recalculated by fixing parameters such as spot position and number of particles in the treatment plan used for each case in clinical practice. This dose was used as the clinical treatment planning reference dose in this study. Second, the CT‐SPR conversion tables for the four conditions, except for the CL, were applied to the same treatment planning CT images, and the doses were recalculated with fixed parameters. Finally, the reference doses for the clinical treatment plan were compared with the doses calculated by applying the CT‐SPR conversion table for the four CT scan protocols. The dose–volume histogram (DVH) parameters of the target and organ at risk (OAR) were evaluated. Among the DVH parameters, relative differences were determined for dose indices (D_mean_, D_xcc_, D_x%_, etc.) using Equation (5). Absolute differences were calculated using Equation (6) for the DVH parameter (e.g., V_x%_) for the volume of the ROI.

(5) $$\mathrm{Relative}\;\mathrm{Difference}\left(\%\right)=\frac{D_{x}-D_{\mathrm{clinical}}}{\mathrm{Prescription}\;\mathrm{Dose}}\times 100$$

(6) $$\mathrm{Absolute}\;\mathrm{Difference}\left(\%\right)=V_{x}-V_{\mathrm{clinical}}$$

D_clinical_ and D_x_ are DVH parameters indicating the dose (e.g., D_mean_, D_xcc_, D_x%_, etc.) when adapting the CT‐SPR conversion tables for the clinical and each CT protocol, respectively. Similarly, V_clinical_ and V_x_ (e.g., V_x%_) are DVH parameters indicating volume when adapting the CT‐SPR conversion tables for the clinical and each CT protocols, respectively. The CIRT treatment plans for 10 cases each of prostate cancer, postoperative pelvic recurrence of colorectal cancer, parotid cancer, and maxillary sinus cancer were used for validation. The study was approved by the Institutional Review Board (2024‐68). Treatment planning CT was obtained with the same CT imaging protocol as Body CL for prostate and postoperative pelvic recurrence of colorectal cancer cases and Head CL for parotid and maxillary sinus cancer cases. All treatment plans were created using RayStation 10A (RaySearch Laboratories, Stockholm, Sweden), using the pencil beam algorithm for physical dose calculations and the modified microdosimetric kinetic model (mMKM) for biological dose calculations.^23^, ^24^ The doses presented in this manuscript were relative biological effective‐weighted doses calculated by mMKM. The irradiation device was a CI‐1000 (Toshiba Energy Systems and Solutions, Kawasaki, Japan) with raster scanning irradiation.^25^, ^26^ The beam angle was set assuming a rotating gantry.^27^ A skilled radiation oncologist with at least 10 years of experience performed target and OAR contouring. The prescribed dose in the treatment plan for all patients with prostate cancer was 51.6 Gy/12 fr.^4^ The prostate and seminal vesicle bases were defined as the clinical target volume (CTV), and the following margins were added to the CTV as planning target volume (left: 10 mm, right: 10 mm, anterior: 10 mm, posterior: 5 mm, superior: 5 mm, inferior: 5 mm). The OAR was defined as the rectum and bladder. The number of beams was two ports for each of all cases. In the treatment planning for patients with postoperative pelvic recurrence of colorectal cancer, CTV1 was defined as the local tumor area plus the prophylactic area and CTV2 as the local tumor area only. The prescribed doses were 41.4 Gy/9 fr for CTV1 and 32.2 Gy/7 fr for CTV2.^28^, ^29^ The bladder, the entire gastrointestinal tract, and peripheral nerves adjacent to the CTV were designated as the OARs. The number of beams in the treatment plan was four or five ports. The prescribed dose in the treatment plan for all parotid cancer cases was 64 Gy/16 fr.^30^ The CTV was set based on the tumor spread relative to the gross tumor volume (GTV). The brain, jawbone, mucous membrane, and skin were defined as OARs. The number of beams was two or three ports. In a case of maxillary sinus cancer, the 64 Gy/16 fr protocol was used for squamous cell carcinoma (two cases), malignant melanoma (six cases), and adenoid cystic carcinoma cases (one case),^31^ while the 70.4 Gy/16 protocol was used for sarcoma case (one case).^32^ In addition to the GTV, the CTV was defined as the area of expected cancer extension. The brain, brainstem, optic chiasma, eye ball, optic peripheral nerve, and skin were defined as OARs. The number of beams was two or four ports. Robust optimization based on mini–max optimization was applied to all treatment plans.^33^ The setup error for each direction in the robust optimization setting was 0.0 cm for right–left, 0.2 cm for superior–inferior, and 0.2 cm for anterior–posterior for the prostate cancer treatment plan and 0.2 cm for all directions for the treatment plan for other cases. Miyasaka et al. found that the error of the CT‐SPR conversion table was <2% based on the results of particle range measurements for similar methods and similar CT scanner combinations. Considering these results, we set a value of 2% for range uncertainty in all cases in our treatment planning. The Statistical Package for the Social Sciences version 28 (IBM SPSS Statistics for Windows; IBM Corp., Armonk, New York, USA) was used for the statistical analysis. The Friedman and Bonferroni tests were used to evaluate significant differences because normality could not be confirmed by the Shapiro–Wilk tests in the comparison. A *p*‐value of <0.05 was accepted as indicating statistical significance. In addition to the evaluation of DVH parameters, a gamma analysis was performed to compare the reference dose of the clinical treatment plan with the plan recalculated by applying the CT‐SPR conversion table for each condition. The tolerances for gamma analysis were 3%/3 mm, 2%/2 mm, and 1%/1 mm. Gamma analysis was set as the global maximum, with a threshold of >10% as the evaluation area. Gamma analysis was performed in 3D gamma analysis using 3D slicer software (https://www.slicer.org/) that can be used for image processing and image analysis. Differences in dose distribution profiles were evaluated to assess the influence of CT imaging protocols on the particle range. Profiles in isocenter were compared in one beam of three cases at each site. Profile comparisons were evaluated by the difference in profiles at the 50% dose position of the prescribed dose.

## 3 RESULTS

### 3.1 CT‐SPR conversion table for each CT scan protocol

**FIGURE 1:**
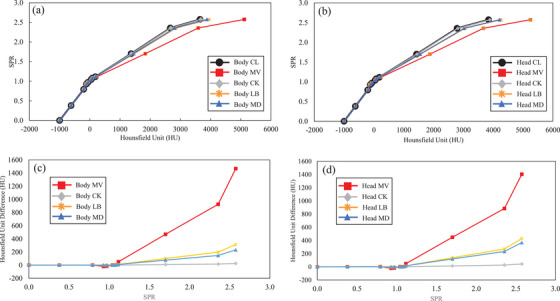
CT‐SPR conversion table curve and CT‐SPR conversion table difference. (a) and (b) show the CT‐SPR conversion tables for Body and Head, respectively, and (c) and (d) show the difference between the CT‐SPR conversion tables for CL and each protocol in Body and Head. CT, computed tomography; SPR, stopping power ratio.

Table 2 presents the HUs for each material obtained under each protocol. The differences between CL and each protocol were generally similar for both the body and head. Body MV exhibited a particularly large difference in the 40% K2HPO4 solution (a difference of approximately 200 HU). The differences between CL and CK were generally small, with a maximum difference of approximately 10 HU. Comparing LB and MD, the difference between CL in air and 40% K2HPO4 solution tended to be larger in LB than in MD. Table 3 presents the HUs of 11 materials derived from the HUs of each material obtained using the method of Kanematsu et al.^13^ In addition, Figure 1 shows the CT‐SPR curves and Hounsfield unit differences for each protocol. In the region of CT values below Muscle/General, which is <100 HU, the differences were small, and for most materials, the differences were within approximately 10 HU. In contrast, the difference tended to increase with higher HUs. The largest difference from CL was in MV, with a difference of >1500 HU in Hydroxyapatite. Differences between CK and CL were small (approximately 50 HU), even for materials with high HUs. For high CT materials, the difference with CL was slightly larger for LB than for MD.

**TABLE 3:** Hounsfield Units of 11 materials calculated based on the polybinary tissue model in each CT.

	Protocol				
Material	Body CL (HU)	Body MV (HU)	Body CK (HU)	Body LB (HU)	Body MD (HU)
Air	−1000.0	−1000.0	−1000.0	−1000.0	−1000.0
Lung	−619.1	−618.2	−619.1	−618.9	−618.8
Extra lung	−204.1	−202.1	−204.1	−203.6	−203.4
Fat	−110.1	−130.2	−111.1	−114.2	−108.8
Adipose/Marrow	−59.8	−74.8	−60.5	−62.8	−58.6
Muscle/General	44.0	46.0	43.9	44.4	45.0
Miscellaneous	81.7	83.9	81.6	82.2	83.1
Heavy spongiosa	183.6	235.0	184.2	194.7	194.3
Mineral Bone	1368.4	1838.0	1376.8	1468.7	1443.9
Tooth	2667.0	3592.4	2683.7	2864.6	2814.5
Hydroxyapatite	3650.2	5118.8	3676.9	3963.7	3882.8

	Protocol				
Material	Head CL (HU)	Head MV (HU)	Head CK (HU)	Head LB (HU)	Head MD (HU)
Air	−1000.0	−1000.0	−1000.0	−1000.0	−1000.0
Lung	−619.2	−618.4	−619.2	−618.9	−618.7
Extra lung	−204.2	−202.5	−204.3	−203.7	−203.3
Fat	−117.5	−139.6	−120.7	−124.7	−117.4
Adipose/Marrow	−65.5	−82.1	−68.0	−70.9	−65.2
Muscle/General	43.5	45.0	43.1	43.9	44.8
Miscellaneous	80.9	82.4	80.4	81.3	82.6
Heavy spongiosa	187.9	235.4	187.9	202.2	203.6
Mineral Bone	1430.0	1878.0	1442.9	1566.9	1548.7
Tooth	2789.7	3673.6	2816.1	3060.0	3022.2
Hydroxyapatite	3846.3	5249.8	3889.0	4275.5	4213.7

Abbreviations: CT, computed tomography; HU, Hounsfield unit.

### 3.2 Treatment plan comparison

**FIGURE 2:**
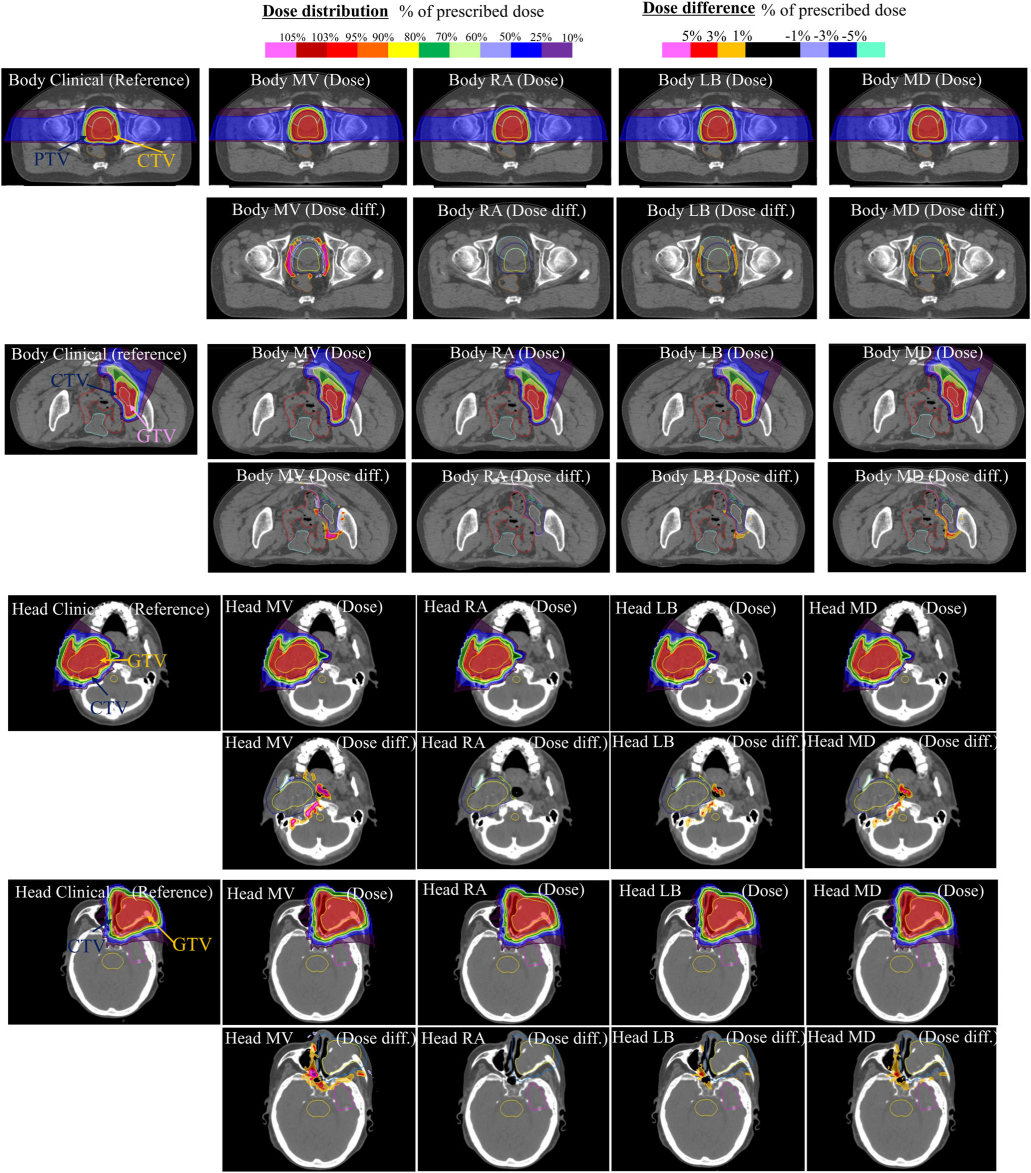
Dose distributions of the clinical treatment planning reference dose and dose calculated by CT with each four CT scan protocol CT‐SPR conversion table and their difference distributions in prostate and postoperative pelvic recurrence of colorectal cancer cases. CT, computed tomography; SPR, stopping power ratio.

**FIGURE 3:**
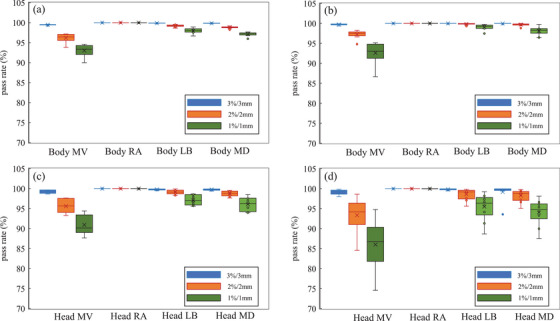
Gamma pass rates for each criterion comparing clinical treatment planning reference dose and dose calculated by CT with each four CT scan protocol CT‐SPR conversion table. (a), (b), (c), and (d) show the results for prostate cancer, postoperative pelvic recurrence of colorectal cancer and parotid cancer cases maxillary sinus cases, respectively. CT, computed tomography; SPR, stopping power ratio.

**FIGURE 4:**
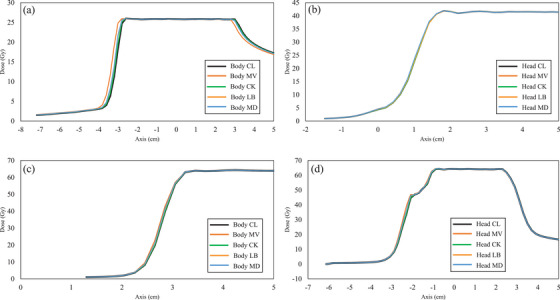
Dose profiles of representative cases. (a), (b), (c), and (d) represent prostate case, postoperative pelvic recurrence of colorectal cancer case, parotid cancer case, and maxillary sinus cancer case, respectively.

Table 4 presents the DVH parameter differences for dose recalculation when the CL and each of the four protocols are applied to the treatment planning CT. Generally, there were no parameters for which the mean difference exceeded 1% for patients with prostate cancer and postoperative pelvic recurrence of colorectal cancer. Among the four protocols, except for the CL, MV tended to have the largest differences exceeding 0.5%, whereas CK had the smallest differences, <0.1% for all parameters. The results of the statistical analysis showed no significant differences in the CK condition except for the GI in the postoperative pelvic recurrence of colorectal cancer cases. LB and MD had approximately the same difference level; however, MD tended to have a slightly larger difference. In the statistical analysis, there were more significant differences in many items in the MD than in the LB. Parotid and maxillary sinus cases generally tended to show greater differences in DVH parameters between CL and each protocol than prostate or postoperative pelvic recurrence of colorectal cancer cases. A mean difference of >1% was observed for MV. The trend of differences in each condition was generally similar to that observed in prostate and postoperative pelvic recurrence of colorectal cancer cases. In the statistical analysis, there were fewer parameters that showed significant differences in the treatment plan for the head and neck region versus the pelvic region. Figure 2 shows dose distribution examples and dose difference maps. In the Figure 2, hot colors were displayed in the distal end region of the dose distribution, indicating that the dose was higher when each of the four conditions was applied than when the clinical protocol was applied. In other words, this result means that the displacement in the direction of range expansion was confirmed under the conditions in which the four CT‐SPR conversion tables were applied, with the exception of the clinical conditions. Differences were observed in the distal fall‐off region among all treatment plans. The dose difference was largest for MV, and almost no difference was observed for CK. Parotid and maxillary sinus cancer cases differed over a wider area and had larger dose differences than prostate cancer and postoperative pelvic recurrence of colorectal cancer cases. Figure 3 presents the results of the gamma analysis between the plans for CL and the dose distribution when each condition CT‐SPR conversion table is applied. The MV condition had the largest difference, with an average gamma passing rate of <80% at 1%/1 mm. For CK, there was almost no difference, and the pass rate was almost 100% even at 1%/1 mm. For LB and MD, the pass rate was almost 100% at 3%/3 mm; however, at 1%/1 mm, the average gamma passing rate decreased to approximately 95%. Table 5 shows the differences in dose profiles from the CL protocol. Furthermore, the dose profiles of the analyzed cases with the largest differences from the CL protocol are shown in Figure 4. Differences in MV tended to be larger than in other parameters. The differences were also larger for MD than for LB.

**TABLE 4:** Differences in DVH parameters clinical treatment planning reference dose and dose calculated by CT with each four CT scan protocol CT‐SPR conversion table.

		Prostate cancer cases							
		Difference (%) [min ‐ max]				*p* (vs. Body CL)			
		Body MV	Body CK	Body LB	Body MD	Body MV	Body CK	Body LB	Body MD
CTV	D_100%_	−0.04 ± 0.11 [−0.31 − 0.14]	−0.01 ± 0.01 [−0.02 − 0.00]	−0.04 ± 0.06 [−0.17 − 0.02]	−0.04 ± 0.05 [−0.17 − 0.02]	1.00	0.81	0.76	0.37
	D_99.5%_	−0.09 ± 0.09 [−0.29 − 0.00]	0.00 ± 0.01 [−0.02 − 0.00]	−0.05 ± 0.05 [−0.16 − 0.00]	−0.05 ± 0.05 [0.00 − 0.04]	0.16	1.00	0.11	0.13
	D_2%_	−1.55 ± 0.43 [−2.13 −0.78]	−0.02 ± 0.06 [−0.19 − 0.00]	−0.41 ± 0.18 [−0.78 − 0.00]	−0.43 ± 0.12[−0.58 −0.19]	<0.01*	1.00	<0.01*	<0.01*
PTV	D_50%_	−0.10 ± 0.04 [−0.17 − 0.00]	0.00 ± 0.00 [0.00 − 0.00]	−0.03 ± 0.01 [−0.04−0.02]	−0.04 ± 0.01 [−0.06 −0.02]	<0.01*	1.00	<0.01*	<0.01*
	D_95%_	−0.60 ± 0.47 [−1.53 − 0.00]	−0.01 ± 0.01 [−0.02 − 0.02]	−0.09 ± 0.07 [−0.21 − 0.00]	−0.13 ± 0.09 [−0.31 −0.02]	0.43	1.00	0.02*	0.02*
	D_99%_	0.12 ± 0.11 [−0.02 − 0.29]	0.00 ± 0.02 [−0.04 − 0.29]	0.02 ± 0.05 [−0.08 − 0.12]	0.02 ± 0.06 [−0.08 − 0.12]	0.10	1.00	1.00	1.00
Rectum	V_30%_	0.08 ± 0.07 [−0.01 − 0.25]	0.00 ± 0.01 [−0.01 − 0.02]	0.01 ± 0.02 [−0.01 − 0.04]	0.02 ± 0.02 [−0.02 − 0.03]	0.12	1.00	0.30	0.02*
	V_40%_	0.03 ± 0.06 [−0.08 − 0.13]	0.00±.000 [−0.00 − 0.0.0]	0.01 ± 0.01 [−0.01 − 0.02]	0.01 ± 0.01 [−0.02 − 0.03]	1.00	1.00	1.00	1.00
	V_50%_	0.07 ± 0.05 [0.00 − 0.20]	0.00 ± 0.00 [−0.01 − 0.01]	0.01 ± 0.01 [−0.01 − 0.02]	0.01 ± 0.01 [−0.01 − 0.03]	0.03*	1.00	0.22	0.30
	V_80%_	0.03 ± 0.02 [−0.01 − 0.06]	0.00 ± 0.01 [−0.01 − 0.02]	0.01 ± 0.01 [−0.01 − 0.03]	0.0.1 ± 0.01 [0.00 − 0.03]	0.01*	1.00	1.87	1.87
	V_95%_	0.00 ± 0.02 [−0.01 − 0.05]	0.00 ± 0.00 [0.00 − 0.01]	0.00 ± 0.01 [−0.02 − 0.01]	0.00 ± 0.01 [−0.01 − 0.01]	1.00	1.00	1.00	1.00
Bladder	V_50%_	0.32 ± 0.21 [0.01 − 0.68]	0.01 ± 0.01 [−0.01 − 0.68]	0.13 ± 0.07 [0.06 − 0.26]	0.19 ± 0.11 [0.08 − 0.44]	0.02*	0.39	0.03*	0.05
	V_95%_	−0.44 ± 0.36 [−1.24 −0.01]	−0.10 ± 0.24 [−0.80 −0.01]	0.17 ± 0.24 [−0.88 −0.01]	−0.20 ± 0.26 [−0.97 −0.05]	0.06	1.00	0.63	0.50

		Postoperative pelvic recurrence of colorectal cancer caces							
		Difference (%) [min ‐ max]				*p* (vs. Body CL)			
		Body MV	Body CK	Body LB	Body MD	Body MV	Body CK	Body LB	Body MD
CTV1	D_98%_	−0.08 ± 0.17 [−0.56 − 0.10]	0.00 ± 0.02 [−0.07 − 0.01]	0.00 ± 0.07 [−0.15 − 0.15]	0.01 ± 0.07 [−0.08 − 0.19]	1.00	1.00	1.00	1.00
	D_95%_	−0.08 ± 0.10 [−0.38 −0.01]	0.00±.00 [0.00 − 0.01]	−0.02 ± 0.02 [−0.07 − 0.00]	−0.01 ± 0.02 [−0.05 − 0.03]	0.47	1.00	0.18	0.81
	D_50%_	−0.01 ± 0.14 [−0.23 − 0.31]	0.00 ± 0.01 [−0.01 − 0.01]	0.01 ± 0.0.5 [−0.05 − 0.12]	0.05 ± 0.13 [−0.01 − 0.43]	1.00	1.00	1.00	1.00
	D_2%_	0.01 ± 0.02 [−0.03 − 0.04]	0.00 ± 0.00 [0.00 − 0.01]	0.00 ± 0.01 [−0.01 − 0.01]	−0.01 ± 0.01 [−0.03 − 0.00]	1.00	1.00	1.00	0.02*
CTV2	D_98%_	−0.12 ± 0.33 [−0.92 − 0.35]	−0.02 ± 0.02 [−0.05 − 0.35]	−0.01 ± 0.07 [−0.12 − 0.11]	0.13 ± 0.18 [−0.05 − 0.58]	1.00	0.18	1.00	0.70
	D_95%_	−0.01 ± 0.06 [−0.15 − 0.08]	0.00 ± 0.01 [−0.03 − 0.01]	0.01 ± 0.02 [−0.03 − 0.05]	0.04 ± 0.08 [−0.04 − 0.23]	1.00	1.00	0.93	1.00
	D_50%_	0.01 ± 0.02 [−0,01 − 0.03]	0.00 ± 0.00 [0.00 − 0.01]	0.00 ± 0.01 [0.00 − 0.01]	−0.01 ± 0.01 [−0.01 − 0.00]	1.00	1.00	1.00	0.13
	D_2%_	0.04 ± 0.12 [−0.08 − 0.37]	0.00 ± 0.01 [[0.00 − 0.01]	0.00 ± 0.02 [−0.03 − 0.04]	−0.03 ± 0.04 [−0.12 − 0.00]	1.00	0.81	1.30	0.32
Bladder	V_30Gy_	0.01 ± 0.27 [−0.44 − 0.51]	0.00 ± 0.01[−0.03−0.01]	0.01 ± 0.07 [−0.11 − 0.13]	0.09 ± 0.09 [0.00 − 0.24]	1.00	1.00	1.00	0.13
	V_50Gy_	−0.04 ± 0.13 [−0.37 − 0.15]	0.00 ± 0.01 [−0.03 − 0.00]	−0.01 ± 0.03 [−0.08 − 0.06]	0.20 ± 0.53 [0.00 − 1.78]	1.00	1.00	1.00	1.00
GI	D_6cc_	0.10 ± 0.12 [−1.21 − 1.02]	−0.06 ± 0.04 [−0.22 −0.01]	0.08 ± 0.18 [−0.22 − 0.31]	0.69 ± 0.26 [0.24 − 1.11]	1.00	0.04*	1.00	<0.01*
	D_2cc_	−0.06 ± 1.21 [−1.68 − 2.30]	−0.10 ± 0.09 [−0.27 − 0.02]	0.01 ± 0.30 [−0.41 − 0.52]	0.78 ± 0.38 [−0.26 − 1.41]	1.00	0.10	1.00	0.02*
Peripheral nerve	D_mean_	−0.04 ± 0.11 [−0.33 − 0.11]	0.00 ± 0.01 [−0.01 − 0.03]	0.01 ± 0.03 [−0.10 − 0.03]	−0.05 ± 0.09 [−0.20 − 0.11]	1.00	1.00	1.00	1.00
	D_1%_	0.07 ± 0.19 [−0.20 − 0.39]	0.00 ± 0.02 [−0.05−0.03]	0.01 ± 0.07 [−0.11 − 0.12]	0.07±.33 [−0.20 − 0.98]	1.00	1.00	1.00	1.00
	D_30%_	0.24±−0.04 [−0.42 − 0.39	0.00 ± 0.01 [−0.01− 0.01]	0.01 ± 0.10 [−0.15 − 0.27]	−0.02 ± 0.17 [−0.38 − 0.27]	1.00	1.00	1.00	1.00
	D_60%_	−0.12 ± 0.25 [−0.56 − 0.45]	−0.05 ± 0.13 [−0.43 − 0.00]	−0.03 ± 0.24 [−0.50 − 0.54]	−0.01 ± 0.18 [−0.26 − 0.45]	1.00	1.00	1.00	1.00
	D_0.01cc_	0.01 ± 0.27 [−0.49 − 0.35]	0.00 ± 0.02 [−0.03 − 0.04]	0.02 ± 0.11 [−0.11 − 0.27]	−0.04 ± 0.21 [−0.49 − 0.37]	1.00	1.00	1.00	1.00

Parotid cancer cases									
		Difference (%) [min ‐ max]				*p* (vs. Head CL)			
		Head MV	Head CK	Head LB	Head MD	Head MV	Head CK	Head LB	Head MD
CTV	D_98%_	−0.16 ± 0.23 [−0.72 − 0.09]	0.00 ± 0.02 [−0.03 − 0.02]	−0.03 ± 0.07 [−0.14 − 0.08]	−0.04 ± 0.07 [−0.17 − 0.08]	0.65	1.00	1.00	0.99
	D_95%_	−0.09 ± 0.10 [−0.33 − 0.05]	0.01 ± 0.02 [−0.03 − 0.03]	−0.03 ± 0.003 [−0.11 − 0.00]	−0.02 ± 0.03 [−0.06 − 0.03]	0.20	1.00	0.35	0.22
	D_50%_	0.00 ± 0.01 [−0.02 − 0.05]	0.00 ± 0.00 [0.00 − 0.00]	0.00 ± 0.00 [0.00 − 0.00]	0.00 ± 0.00 [0.00 − 0.00]	1.00	1.00	1.00	1.00
	D_2%_	0.01 ± 0.03 [−0.03 − 0.06]	0.00 ± 0.00 [0.00 − 0.02]	0.00 ± 0.01 [−0.02 − 0.02]	0.00 ± 0.01 [−0.02 − 0.02]	1.00	1.00	1.00	1.00
Barin	D_mean_	0.15 ± 0.22 [0.00 − 0.77]	0.00 ± 0.00 [0.00 − 0.00]	0.03 ± 0.09 [−0.14 − 0.23]	0.05 ± 0.08 [0.00 − 0.27]	0.64	1.00	1.00	0.64
	D_1cc_	1.53 ± 1.28 [0.02 − 4.38]	0.00 ± 0.05 [−0.09 − 0.08]	0.54 ± 0.09 [0.02 − 1.52]	0.61 ± 0.49 [0.02 − 1.58]	0.06	1.00	0.91	0.05
Jaw bone	D_mean_	−0.06 ± 0.34 [−0.50 − 0.84]	−0.01 ± 0.00 [−0.03 − 0.02]	−0.03 ± 0.12 [−0.19 − 0.30]	−0.01 ± 0.14 [−0.23 − 0.34]	1.00	0.51	1.00	1.00
	D_1cc_	−0.41 ± 0.24 [−0.80 − 0.00]	−0.01 ± 0.03 [−0.06 − 0.02]	−0.10 ± 0.09 [−0.33 − 0.03]	−0.08 ± 0.11 [−0.33 − 0.06]	0.01*	1.00	0.09	0.55
Mucous membrane	D_2cc_	0.43 ± 0.56 [0.00 − 1.94]	−0.04 ± 0.10 [−0.23 − 0.06]	0.13 ± 0.26 [−0l.13 − 0.6]	0.29 ± 0.35 [0.00 − 1.00]	0.77	1.00	1.00	0.35
	D_mean_	0.11 ± 0.27 [−0.11 − 0.81]	−0.03 ± 0.04 [−0.13 − 0.00]	0.06 ± 0.09 [−0.03 − 0.27]	0.15 ± 0.20 [0.00 − 0.62]	0.71	0.79	0.65	0.50
Skin	D_1cc_	0.13 ± 0.33 [−0.47 − 0.81]	0.13 ± 0.34 [−0.48 −0.78]	0.10 ± 0.33 [−0.48 − 0.81]	−0.01 ± 0.23 [−0.05 − 0.55]	1.00	1.00	1.00	1.00

Maxillary sinus cancer cases									
		Difference (%) [min ‐ max]				*p* (vs. Head CL)			
		Head MV	Head CK	Head LB	Head MD	Head MV	Head CK	Head LB	Head MD
CTV	D_98%_	0.25 ± 0.18 [0.06 − 0.66]	−0.02 ± 0.03 [−0.09 − 0.02]	0.16 ± 0.07 [0.06 − 0.27]	0.20 ± 0.11 [0.03 − 0.39]	0.10	1.00	0.15	0.09
	D_95%_	0.07 ± 0.11 [−0.09 − 0.22]	−0.04 ± 0.10 [−0.34 − 0.02]	0.03 ± 0.05 [−0.05 − 0.13]	0.04 ± 0.06 [−0.05 − 0.14]	0.99	1.00	0.24	0.11
	D_50%_	−0.01 ± 0.02 [−0.05 − 0.03]	0.00 ± 0.01 [0.00 − 0.02]	0.00 ± 0.01 [−0.02 − 0.02]	−0.01 ± 0.01 [−0.02 − 0.02]	1.00	1.00	1.00	0.52
	D_2%_	0.05 ± 0.19 [−0.05 − 0.61]	0.00 ± 0.01 [−0.02 −0.02]	0.01 ± 0.04 [−0.03 − 0.11]	0.01 ± 0.05 [−0.03 − 0.14]	1.00	1.00	1.00	1.00
Brain	D_1cc_	0.81 ± 0.82 [−0.55 − 2.08]	0.00 ± 0.04 [−0.08 − 0.05]	0.26 ± 0.33 [−0.36 − 0.75]	0.31 ± 0.36 [−0.38 − 0.94]	0.16	1.00	0.41	0.25
Brainstem	D_0.01cc_	0.97 ± 1.57 [−0.22 − 5.25]	−0.04 ± 0.05 [−0.13 − 0.05]	0.29 ± 0.49 [−0.08 − 1.64]	0.43 ± 0.42 [−0.02 − 1.58]	0.95	0.34	1.00	0.28
Chiasma	D_0.01cc_	1.05 ± 0.96 [0.22 − 3.24]	0.03 ± 0.21 [−0.09 − 0.66]	0.45 ± 0.45 [−0.14 − 1.30]	0.65 ± 0.51 [−0.14 − 1.39]	0.10	1.00	0.15	0.90
EyeBall_ipsilateral	D_1cc_	0.07 ± 0.34 [−0.41 − 0.88]	−0.01 ± 0.03 [−0.08 − 0.03]	−0.01 ± 0.11 [−0.22 − 0.36]	0.03 ± 0.20 [−0.25 − 0.48]	0.17	0.52	0.26	0.40
EyeBall_contrateral	D_1cc_	0.40 ± 0.68 [−0.20 − 2.00]	−0.04 ± 0.06 [−0.17 − 0.02]	0.12 ± 0.19 [−0.09 − 0.58]	0.21 ± 0.27 [−0.11 − 0.70]	0.14	0.24	0.10	0.01*
OpticNerve_ipsilateral	D_0.1cc_	0.52 ± 1.04 [−0.48 − 2.58]	0.00 ± 0.06 [−0.09 − 0.13]	0.09 ± 0.40[−0.23 − 1.14]	0.15 ± 0.46 [−0.30 − 1.16]	1.00	1.00	1.00	1.00
OpticNerve_contrateral	D_0.1cc_	1.55 ± 1.79 [−0.37 − 6.17]	−0.03 ± 0.07 [−0.19 − 0.03]	0.59 ± 0.46 [−0.08 − 1.55]	0.62 ± 0.68 [−0.17 − 1.95]	0.03*	0.48	0.01*	0.01*
Skin	D_1cc_	0.07 ± 0.07 [0.00 − 0.22]	0.01 ± 0.01 [0.00 − 0.03]	0.02 ± 0.03 [0.00 − 0.11]	0.00 ± 0.02 [−0.03 − 0.03]	0.12	0.24	0.45	1.00

Abbreviations: CT, computed tomography; CTV, clinical target volume; DVH, dose volume histogram; Dmean, mean dose; D_x%_, minimum dose to the most irradiated x% of tissue volume; D_xcc_, minimum dose to the most irradiated xcc of tissue volume; PTV, planning target volume; GI, gastrointestinal; SPR, stopping power ratio; V_x%_, the ratio of the volume irradiated by x% or more of the prescribed dose; V_xGy_, the ratio of volume irradiated with xGy or more.

**TABLE 5:** Differences in dose profiles from CL protocol.

	Dose profile difference (mm) [mean ± 1SD]			
	MV	CK	LB	MD
Prostate cancer case	1.4 ± 0.6	0.1 ± 0.1	0.4 ± 0.2	0.5 ± 0.2
Postoperative pelvic recurrence of colorectal cancer case	0.1 ± 0.0	0.0 ± 0.0	0.0 ± 0.0	0.1 ± 0.1
Parotid cancer case	0.1 ± 0.1	0.0 ± 0.0	0.0 ± 0.0	0.1 ± 0.1
Maxillary sinus cancer cases	0.6 ± 0.4	0.0 ± 0.0	0.1 ± 0.2	0.2 ± 0.2

## 4 DISCUSSION

The impact of different treatment planning CT scan protocols and CT models on treatment planning in CIRT was evaluated. In the past, Davis et al. summarized dose changes with CT scan protocols for X‐ray therapy in a review.^34^ They reported that the maximum difference margin for any change in CT scan protocol is 1%–2%. In contrast, the CIRT used in this study showed an average difference of <1% and a maximum difference of approximately 4%, which is similar to or larger than that of the X‐ray treatment. The difference in dose gradients may be related to these effects. The dose gradients for the equipment at our facility were 80%–20% of the prescribed dose in the beam model, with a transverse penumbra of 7.7 mm for the carbon ion beam and 22.1 mm for the photon beam. Furthermore, the distance required to decrease from 80% to 20% of the dose in the CIRT distal fall‐off region was 5.1 mm. This means that a 1% diffenrece in the SPR calculation will result in a difference of approximately 25% in the point dose in the distal fall‐off region. This steep dose gradient can be attributed to the large differences in CIRT.

Each difference occurred at the distal end of the dose distribution. The trend was that when the CT‐SPR conversion table for each protocol was applied, there was a change in the range extension relative to the range calculated by applying the clinical protocols. In the change of HU in each protocol, the change of HU of K2HP4 was especially large among the materials in the phantom, and the HU was high for the clinical protocol. The change in HU, as identified in this study, is in the direction of increasing range, and the data in this study revealed the effect on range for a change in HU.

In the present validated CT scan protocols, the effects of tube voltage were the most significant, changing the HU of the high SPR material by approximately 200 HU. The variation in HUs with tube voltage has been described in previous reports and was similar in this study.^35^ The differences in dose and dose distribution profiles resulting from the effects of the range calculation were the largest among the parameters considered in this study. Therefore, changes in tube voltage are considered to be one of the most important considerations among the numerous CT scan parameters.

Davis et al. reported the effects of the convolution kernel on HUs.^20^ According to the report, the difference between FC13 and FC64 considered in this study was reported to be approximately 45 HU at most and almost no difference depending on the materials. In contrast, the differences in this study were also small, with HUs <10 HU, confirming a similar trend. The different convolution kernel modified in this study resulted in changes in the correction for beam hardening. Marques et al. reported that the effects of beam hardening correction on CT images were changes in spatial resolution and HUs.^21^ In our study, the change in HU was also observed; however, the change in DVH evaluation relative to dose was 0.1%. The influence of the convolution kernel is therefore considered to be minimal in the treatment plan, and the selection of the convolution kernel should be considered considering image quality and contouring accuracy.

According to site, differences were larger in the head and neck region, particularly in maxillary sinus cases. The maxillary sinus area has a complex bony structure, which may be the reason for the large difference in HUs due to the difference in CT scan protocols. Furthermore, the OARs were present in close proximity to the tumor around the paranasal sinuses, and the slight difference in range may have resulted in a difference in OAR doses.

In this study, differences in treatment planning doses were caused by differences in the CT scanners and CT models. Peter et al. compared CT‐SPR conversion tables for each CT scanner in European proton facilities. They mentioned the effects of beam hardening as a factor that affected the CT‐SPR conversion table. Furthermore, differences in X‐ray energy and HUs between different models have been reported,^36^, ^37^ suggesting the need for calibration for each CT scanner model. The results of this study highlight the need for calibration of each CT scanner and reveal the impact of calibration tables.

The gamma analysis results revealed that the gamma pass rate was close to 100% at 3%/3 mm under all conditions; however, the pass rate decreased significantly from 2%/2 mm to 1%/1 mm. This suggests that most differences in dose from CT scan protocols are in the range of 1–2 mm and 1%–2%. Similar to the evaluation of DVH parameters, the gamma pass rate tended to be lower in parotid and maxillary sinus cases than in prostate cancer and postoperative pelvic recurrence of colorectal cancer cases, indicating that the head and neck region was more affected than the pelvic region.

Taylar et al. examined appropriate phantom materials for carbon beams.^38^ They noted that while some commercial phantoms are appropriate for CIRT quality assurance, there is individual variability in low‐density materials and care should be taken in handling them. We used water, ethanol, and K2HPO4 solutions as our materials, so we believe our results are reproducible and not biased by the materials used. However, the use of solid phantoms or low‐density materials to validate our results would need to be carefully considered in light of the material's validity as a water equivalent or tissue equivalent.

Adaptive radiotherapy^39^, ^40^ and adaptive particle therapy^41^, ^42^ are now being used in clinical practice. This means that treatment planning is performed on multiple CT systems other than the CT for treatment planning. Under such an operation, it is anticipated that a single common CT‐SPR conversion table may be used at the facility to simplify the QA and treatment planning process. The results of this study provided quantified data on the impact of CT values on treatment planning in patients undergoing this type of treatment.

This study has several limitations. First, the results of this study are only simulation results. We have not validated the results of this study using dosimetry. Miyasaka et al. reported that the comparison between actual measurements and measurements under the clinical protocol in this study was approximately 2%,^10^ which is an acceptable error for clinical use, confirming the results for clinical conditions; however, further measurements are necessary for other scan protocols for comparison. Second, this study was limited to single‐energy CT. Furthermore, the accuracy of CT‐SPR conversion tables can be improved by dual‐energy^43^ and proton CT.^44^, ^45^ Detailed comparisons using such techniques are necessary to improve the accuracy of SPR estimation in treatment planning. Also, in this study we did not evaluate the treatment of areas with respiratory motion, such as the lungs and liver. Additional imaging, such as four‐dimensional CT (4DCT), is needed for treatment planning to account for respiratory movement, and additional studies are needed. Because these additional considerations would have complicated our understanding of the data and made our conclusions difficult to understand, in this study we only evaluated areas without respiratory movement. Further studies are needed to evaluate the use of 4DCT in areas with respiratory movement.

## 5 CONCLUSION

This study evaluated the impact of differences in HUs and CT‐SPR conversion tables caused by differences in CT scan protocols and CT scanners on treatment planning. As a result, an average dose difference of about 1% and a maximum difference of about 5% were confirmed. In particular, it was confirmed that the effect of tube voltage variation is a parameter that requires significant attention. The impact of changes in the reconstructed kernel is small, and selection of an appropriate convolution kernel is recommended. Differences of more than 1% were observed even for different scanner and model, which clearly shows the importance of calibration for each device.

## AUTHOR CONTRIBUTIONS

**Yuya Miyasaka**: Conceptualization, writing—original draft and investigation. **Hikaru Souda**: Validation and writing—review and editing. **Yasuhito Hagiwara**: Contouring ROIs, clinical review, and review manuscript. **Hiroko Akamatsu**: Contouring ROIs, clinical review, and review manuscript. **Mayumi Harada**: Contouring ROIs, clinical review, and review manuscript. **Takashi Kaneko**: Contouring ROIs, clinical review and review manuscript. **Hongbo Chai**: Validation, review, and editing. **Miyu Ishizawa**: Validation and writing—review and editing. **Hiraku Sato**: Contouring ROIs, clinical review, and review manuscript. **Masashi Koto**: Contouring ROIs, clinical review, and review manuscript. **Takeo Iwai**: Project administration and writing—review and editing.

## CONFLICT OF INTEREST STATEMENT

The authors declare that they have no conflict of interest.

## Data Availability

Research data are stored in an institutional repository and will be shared upon request to the corresponding author.
